# Supplementary material for: Shared Treg dysregulation underlies psoriasis vulgaris and atopic dermatitis through the PHF19-PRC2 epigenetic axis
Source: Front Immunol. 2026 Aug 20;17:1932788. doi: 10.3389/fimmu.2026.1932788 (PMC13538097; doi:10.3389/fimmu.2026.1932788)
Supplement: Supplementary file 3 [file DataSheet3.zip › Additional files/Additional file 4/56.HLA-DRB5.leaveoneout.pdf]

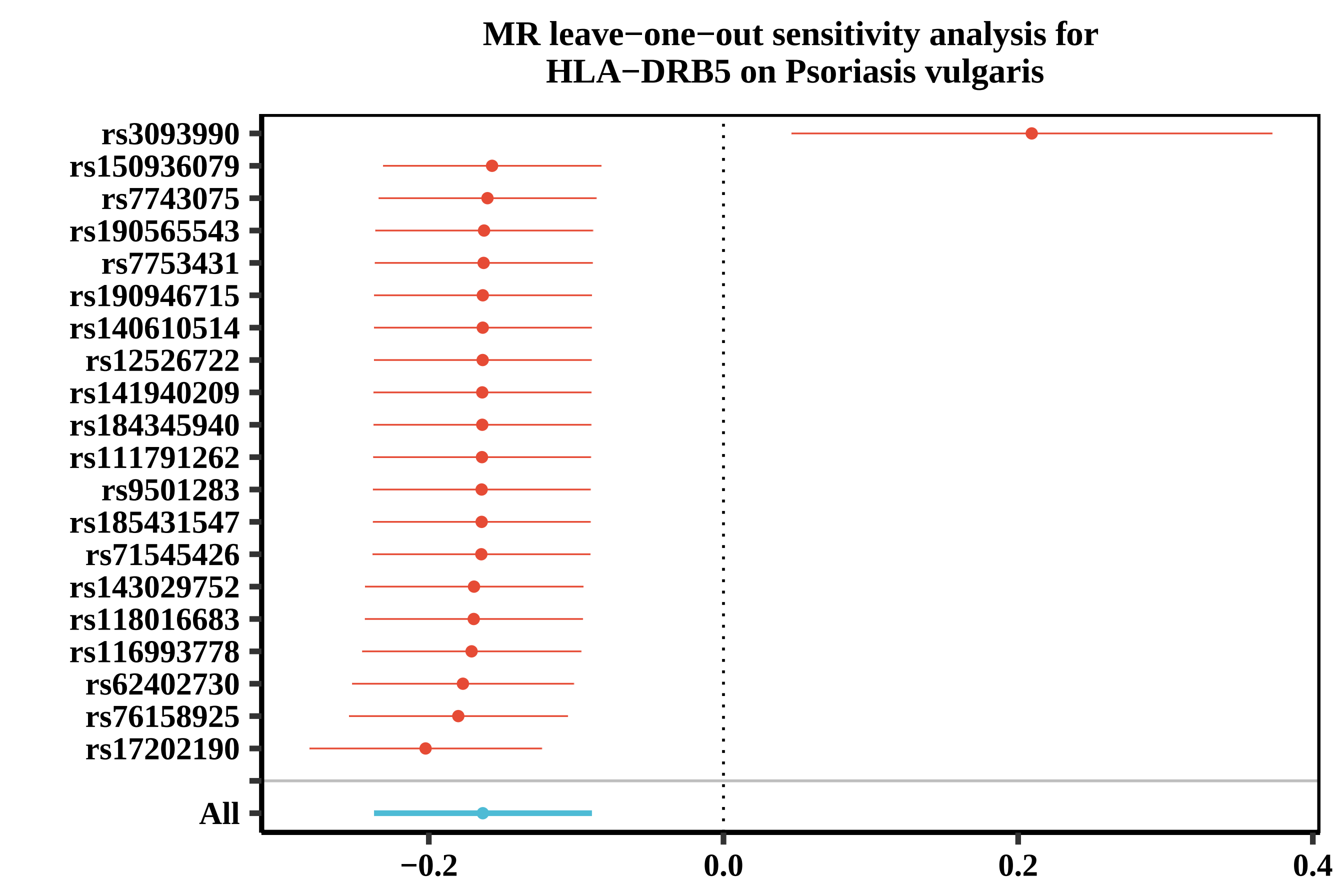
MR leave−one−out sensitivity analysis for
HLA−DRB5 on Psoriasis vulgaris
rs3093990
rs150936079
rs7743075
rs190565543
rs7753431
rs190946715
rs140610514
rs12526722
rs141940209
rs184345940
rs111791262
rs9501283
rs185431547
rs71545426
rs143029752
rs118016683
rs116993778
rs62402730
rs76158925
rs17202190
All
−0.2
0.0
0.2
0.4
